# Let Food Be Thy Medicine—Its Role in Crohn’s Disease

**DOI:** 10.3390/nu13030832

**Published:** 2021-03-03

**Authors:** Judith Wellens, Séverine Vermeire, João Sabino

**Affiliations:** Department of Gastroenterology and Hepatology, University Hospitals Leuven, 3000 Leuven, Belgium; Judith.wellens@kuleuven.be (J.W.); severine.vermeire@uzleuven.be (S.V.)

**Keywords:** Crohn’s disease, inflammatory bowel diseases, nutritional therapy, diet, nutrition

## Abstract

The food we eat is thought to play a role in both the increasing incidence as well as the course of Crohn’s disease. What to eat and what to avoid is an increasingly important question for both patients and physicians. Restrictive diets are widely adopted by patients and carry the risk of inducing or worsening malnutrition, without any guarantees on anti-inflammatory potential. Nevertheless, exploration of novel therapies to improve long-term management of the disease is desperately needed and the widespread use of exclusive enteral nutrition in the induction of paediatric Crohn’s disease makes us wonder if a similar approach would be beneficial in adult patients. This narrative review discusses the current clinical evidence on whole food diets in achieving symptomatic and inflammatory control in Crohn’s disease and identifies knowledge gaps with areas for future research.

## 1. Introduction

Inflammatory bowel diseases (IBD) are chronic inflammatory conditions of the gastrointestinal tract encompassing two main clinical entities: Crohn’s disease (CD) and ulcerative colitis (UC) [1,2]. Both conditions are thought to originate in genetically susceptible individuals exposed to certain, unknown environmental risk factors [1]. There is no cure for either disease, but a variety of classic and more innovative oral and biologic therapies are nowadays available with good safety and tolerability profiles [3]. Unfortunately, both primary and secondary loss of response to biologic therapy is substantial, and many patients still lack effective treatment options [4].

Epidemiologically, migration studies show that when moving from low-incidence areas to high-incidence areas, the incidence of IBD for the second generation resembles that of the resident population [5,6]. A common environmental suspect is the Western-style diet rich in sugar, fat, and protein which has a strong and rapid influence on the intestinal microbiome when compared to a plant-based diet [7]. Translating diet in clinical applications, there is convincing evidence for diet-based therapeutic strategies in paediatric CD, in whom exclusive enteral nutrition is as effective as corticosteroids to induce clinical and endoscopic remission and is, therefore, commonly used as a first-line therapy; however, long-term adherence is challenging, and disease recurrence after a regular diet is resumed, is to be expected [8,9,10,11,12,13].

The question: ’Doctor, what should I eat?’, is very common and forces doctors to advise a ‘healthy diet’ or ‘eat what you can tolerate’ due to lack of robust evidence [14,15,16]. In spite of this, evidence from surveys shows that the majority of IBD patients reports specific changes in their diet believing in beneficial effects on symptom severity and inflammatory episodes [17,18]. The choice of which foods to avoid is generally based upon their own recognition of adverse symptoms, or upon advice given externally through other patients or popular diet books [17,18,19]. Importantly, since patients with IBD already are at increased risk of malnutrition and weight loss, the possible effects of a restrictive diet should be assessed and monitored closely by a dietitian or a physician [16]; so are 40–78% of IBD patients believed to have inadequate folate intake, which is predominantly found in (leafy) vegetables [18,20,21]. The avoidance of fruits and vegetables can be so strict, that patients may develop symptoms of scurvy due to vitamin C deficiency [22]. Additionally, 80–86% of IBD patients are reported to have inadequate dietary intake of calcium, possibly as a result of avoiding milk and dairy, which were also identified as foods thought to worsen symptoms [18,23].

Although nutrition seems to be a key influencer of disease pathogenesis, up to now doctors have been struggling to give evidence-based dietary advice. This leads to the adoption of various -often restrictive and unsupervised- diets by patients based on symptomatic control, while the aim of therapeutic management is to achieve and maintain remission and prevent disease progression [24,25]. Given this gap between patient wishes and physician’s knowledge on dietary guidance, we felt the need for this current update on dietary knowledge in CD.

## 2. Methods

We aimed to narratively review the current literature of dietary strategies in patients with Crohn’s disease (both adults and children), in management of both inflammatory and symptomatic control. Therefore, we gathered high-quality papers including meta-analyses, systematic reviews, randomized controlled trials, and observational data to summarize the role of different diets. Since exclusive enteral nutrition in paediatric Crohn’s disease is already extensively reviewed elsewhere, this is outside the scope of this review. Symptomatic control was considered as any improvement of quality-of-life scores, clinical remission (scores) or merely when patients reported ‘improvement of symptoms’. Inflammatory responses comprise endoscopic improvement, mucosal healing, and biochemical responses measured by C-reactive protein (CRP), erythrocyte sedimentation rate (ESR), faecal calprotectin, cytokines such as interleukin-6 (IL-6), serum albumin, heamatocrit and platelet count.

We included strategies aimed at inducing or maintaining remission of Crohn’s disease and/or affecting abdominal symptoms and/or quality of life. Only validated scoring systems or endoscopic results were deemed acceptable. Studies aimed at reducing post-operative recurrence were included as well. Only papers with a full text available, written in English and published in the last 20 years were included to get a contemporary and robust overview.

We excluded in vitro/ex vivo studies, animal studies, letters to the editor, editorials, case reports, narrative reviews, opinions, comments and poster presentations. Furthermore, studies involving strategies concerning antibiotics, medical therapy (only), parenteral nutrition, enteral nutrition as an induction therapy in paediatric patients and peri-operative management as their primary endpoint were also excluded. Studies primarily aimed at studying the epidemiology of Crohn’s disease, non-abdominal symptoms (e.g., arthralgias) or other end points (e.g., bone mineral density, levels of vitamin D, …) were not retained.

## 3. Overview of Different Dietary Strategies

### 3.1. EEN (Exclusive Enteral Nutrition) and PEN (Partial Enteral Nutrition)

Enteral nutrition is a mode of nutritional therapy in CD in which all (exclusive enteral nutrition, EEN) or some (partial enteral nutrition, PEN) of the total calories are derived from a liquid formula-based diet delivered to the gastro-intestinal tract orally or through nasogastric feeding (Table 1) [26]. Enteral formulas are classified into 3 types: (1) polymeric formulations that contain intact proteins, carbohydrates and fats; (2) semi-elemental formulas that contain peptides of varying chain lengths, simple sugars, glucose polymers or starch, and fat, primarily as medium-chain triglycerides; and (3) elemental formulations that contain partially or completely hydrolysed nutrients (shorter-chain amino acids and fatty acids with only 2% to 4% long-chain triglycerides) [16]. A Cochrane meta-analysis by Narula et al. in 2018 could not demonstrate any significant benefit based on the formula composition but clearly showed effectiveness of EN for the induction of remission in adult patients with CD [26]. Supporting this statement, a prospective trial in adult patients by Yamamoto et al. in 28 active CD patients showed that after 4 weeks of EEN, 71% of patients achieved clinical remission, with endoscopic healing in 44% in the terminal ileum and 39% in the large bowel. Histologic healing was observed in 19% in the terminal ileum and in 20% in the large bowel [27]. Yang et al. prospectively showed in 41 patients that EEN during 12 weeks was effective in inducing early clinical remission, mucosal healing, promoting fistula closure and reducing the size of abscesses [28].

Because EEN cannot be used indefinitely, a maintenance strategy has to be sought. PEN, where typically only 25–50% of the total calories are administered using a formula, has been studied extensively over the last 20 years in 14 studies (3 randomized controlled trials (RCTs), 6 retrospective, and 5 prospective studies) in both adults and children with CD of which only 2 of them were negative [29,30,31,32,33,34,35,36,37,38,39,40,41,42]. In summary, one can appreciate an overall beneficial effect of PEN on relapse rates to some extent, although the formulas used, type of induction, control groups (PEN but lower number of calories or habitual diet), and length of the intervention differed greatly among the studies.

Three studies have investigated the effect of EN on disease recurrence after (ileocolonic) resection and anastomosis with encouraging results. As an extension of a previous positive study with 1-year follow-up, the data after 5 years in 40 adult patients showed a persistently lower cumulative recurrence incidence rate requiring infliximab in the EN group vs. the control group (*p* = 0.02). The cumulative incidence of reoperation was also lower in the EN group vs. the control group, although the difference was not significant [41,43]. In addition, Ohara et al. concurred with this beneficial effect in improving symptomatic and endoscopic recurrence-free duration in their 2-year retrospective analysis in 38 patients [34].

Taken together, in terms of efficacy, enteral nutrition (exclusive or partial) seems a valuable tool in induction (and maintenance) of remission, especially in luminal and ileocolonic disease [44].

### 3.2. SCD (Specific Carbohydrate Diet)

The specific carbohydrate diet (SCD), developed in the 1920s and popularized at the beginning of the 1940s, restricts complex carbohydrates and eliminates refined sugar from the diet. This is based on the rationale that the sugars and complex carbohydrates are poorly absorbed and could eventuate in bacterial dysbiosis contributing to the intestinal inflammation of IBD [45]. Because of its biological plausibility, the popularity in lay press and adoption by patients, the scientific community responded with formal testing in IBD patients. We reviewed 8 studies performed in the last decade. The first prospective trial was led by Cohen et al. and included 10 children with active CD after patency testing with a capsule [45]. Nine patients made it to the first endpoint at 12 weeks and 7 continued the diet until 52 weeks of follow-up. At 12 weeks they noticed a significant decrease in the Harvey Bradshaw Index (HBI) and Paediatric Crohn Disease Activity Index (PCDAI), and this improvement persisted at 52 weeks. As for endoscopic endpoints, the Lewis score (LS) decreased significantly after 12 weeks and sustained endoscopic improvement was seen by week 52. Moreover, 2 patients showed sustained mucosal healing [45].

Secondly, a small retrospective study in 7 CD children from Suskind and colleagues showed encouraging results with a sustained resolution of symptoms in all patients at a routine clinic visit 3 months after initiating the diet [46]. Laboratory indices, including serum albumin, CRP, haematocrit, and stool calprotectin, either normalized or significantly improved during follow-up clinic visits [46].

Kakodkar et al. performed a survey in 50 adult IBD patients (26 CD) in remission using the SCD diet. Self-reported efficacy using a visual analogue scale was rated as a mean of 91.3% effectivity in controlling acute flare symptoms (30–100%) and a mean of 92.1% effective at maintaining remission (53–100%). Subjects reported a mean of 40% difficulty rating in following the diet (0–100%) [47]. However, caution interpreting the results is warranted given that the study population included only patients already using this diet [47].

Subsequently, a larger web-based questionnaire in 417 IBD patients (47% CD) on the SCD diet by Suskind et al. showed that individuals perceived clinical improvement on the SCD [48]. Four percent reported clinical remission prior to the SCD, while 33% reported remission 2 months after initiation of the SCD, and 42% at both 6 and 12 months. For those reporting clinical remission, 13% reported that the time to achieve remission was less than 2 weeks, for 17% it was 2 weeks to a month, 36% reported 1 to 3 months, and 34% reported greater than 3 months [48]. Again, this was a (web-based) survey and data were not controlled.

Burgis et al. followed 11 paediatric CD patients who initiated the SCD at the time of diagnosis or flare to assess the effectiveness of SCD on maintenance of remission [49]. Although an improvement of haematocrit, albumin and ESR values while on a strict SCD was reported and this appeared stable after liberalization of the diet, both patients on the SCD ‘simple ‘(diet alone, antibiotics or 5-ASA) or SCD with immunomodulators (corticosteroids and/or stable thiopurine dosing) were included, raising the question if these positive results would have been obtained to the same extent when using a control [49].

Moreover, another retrospective study during 1 year in 26 paediatric IBD patients (20 CD) showed that although the clinical situation of the included CD patients varied (e.g., timing of start SCD) in active CD, the PCDAI decreased after implementation of the SCD diet [50].

Considering the more recently applied endpoints of mucosal healing, Wahbeh et al. reported that in 7 asymptomatic CD patients on a ‘modified’ SCD (addition of ‘illegal food’) no patient achieved complete macroscopic mucosal healing of both the ileocolonic and upper gastrointestinal tract [51]. Interestingly, all subjects reported an absence of clinical symptoms and the majority had consistently normal CRP, albumin and haematocrit assessments, and mildly elevated faecal calprotectin at any point within 3 months before the repeat endoscopy [51]. After restaging disease for mucosal healing 5 of 7 patients were started on anti-TNF therapy [51]. Despite the fact that non-adherence to a strict SCD might play a role, these data underscore the need for endoscopic assessment to direct therapeutic decision-making.

Finally, a US prospective trial in 12 paediatric patients with mild to moderate IBD with SCD monotherapy showed a mean PCDAI decrease from 28.1 ± 8.8 to 4.6 ± 10.3 at 12 weeks [52]. Mean Pediatric Ulcerative Colitis Activity Index (PUCAI) decreased from 28.3 ± 23.1 to 6.7 ± 11.6 at 12 weeks. Stool microbiome analysis showed significant changes in microbial composition after the dietary intervention. Thus, clinical and objective laboratory improvements were seen in the majority of patients with many patients achieving clinical remission and normalization of inflammatory markers [52]. These encouraging results nonetheless need confirmation by a larger comparative, controlled trial with a metabolomic approach and endoscopic assessment to ascertain its full clinical applicability.

### 3.3. Immunoglobulin G (IgG)-Guided Exclusion Diet

As food antigens may play a role during the initiation or perpetuation of IBD [53], a personalized dietary strategy based on exclusion of these specific foods has been proposed. The first evidence of possible efficacy was generated by Bentz et al. in a double-blind cross-over trial in 23 CD patients (both active and inactive disease) for 12 weeks. An IgG-specific diet based on IgG reactivity to food antigens was compared to a sham diet that included similar products excluded in the specific diet (e.g., cauliflower-broccoli). They tested 271 antigens, of which high and very high reactivity foods were excluded [53]. Although a significant reduction in stool frequency in the specific diet group was seen during the first 6 weeks, this did not persist beyond this period, questioning the relevance of this results [53].

Another 4-week prospective study by Rajendran et al. in 29 patients with CD showed that exclusion of the 4 most reactive foods in terms of IgG4 response resulted in a symptomatic improvement in 90% of patients assessed by a decrease in modified CD activity index (mCDAI) and a significant decrease in ESR [54]. As a secondary endpoint, the IgG4 titres for the excluded foods fell significantly after exclusion [54].

The first RCT compared a IgG4-guided exclusion diet (exclusion of 4 foods with highest antibodies, 16 food types tested) to a diet excluding the 4 foods with the lowest antibody titres. This 4-week study showed an improvement in quality of life in the interventional diet group [55]. Furthermore, the CDAI and HBI improved significantly, although no significant changes were observed in CRP or faecal calprotectin [55].

Lastly, a prospective cohort study with retrospective control group in adult CD patients in remission after 1–3 months of EEN was conducted by Wang et al. [56]. After the EEN induction, 32 patients received an IgG-guided diet excluding the moderately and strongly immunoreactive foods (of 14 foods tested) and were compared to a habitual diet [56]. Disease relapse occurred in 12.5% (4/32) in the exclusion diet group compared to 25% (8/32) in the control group with a significant decrease in CDAI and ESR. These results were not paralleled by similar decreases in CRP, nor in the Simple Endoscopic Score for Crohn’s Disease (SES-CD) [56].

Taken together, although there are signs of symptomatic benefit after adopting a personalised diet based on IgG-titers, these data do not support this strategy when pursuing reduction of inflammatory burden in CD.

### 3.4. CDED (Crohn Disease Exclusion Diet)

The Crohn Disease Exclusion Diet (CDED) is a whole food diet that can be combined with PEN. Specifically, gluten, dairy products, gluten-free baked goods and breads, animal fat, processed meats, products containing emulsifiers, canned goods, and all packaged products with a due date are not allowed during the initial period. It does allow specific spices and herbs, whereas all other condiments and sauces are not permitted. Up to 18 to 20 g of fibre per day can be consumed. In the second 6-week period, a fixed portion of whole grain bread is allowed as are small amounts of nuts, fruits, legumes and vegetables. Patients with strictures should continue quantitative restriction of fruits and vegetables on an individual basis [57].

The first prospective study testing this diet as an induction therapy included 47 patients with active CD (13 adults) starting with CDED and PEN (max 50% of total calories, after 6 weeks only 25% of calories) or CDED monotherapy. After 6 weeks, clinical response and remission were obtained in 78.7% and 70.2% of patients, respectively. The majority of patients who used CDED as monotherapy reached clinical remission [57].

In a retrospective study from the same group including 21 patients (11 adults and 10 children) with loss of response to a biological, clinical remission after 6 weeks of CDED was seen in 61.9% of patients. A significant decrease was seen in HBI and CRP [58].

More recently, a multicentre, open-label RCT of 12 weeks including 78 children with mild-to-moderate CD was performed [10]. In this study, CDED plus 50% PEN for 6 weeks followed by CDED with 25% PEN from weeks 7 to 12 was compared to EEN for 6 weeks followed by a free diet with 25% PEN from weeks 7 to 12. Greater tolerance and corticosteroid-free remission was observed more frequently in the CDED group. Moreover, in children given CDED plus PEN, corticosteroid-free remission was associated with sustained reductions in inflammation (based on CRP and faecal calprotectin) [10].

Together, these data suggest that CDED might be an attractive therapeutic option for CD patients. Larger efficacy powered studies are needed to confirm these observations.

### 3.5. CD-TREAT (Treatment-with-Eating Diet)

The Crohn’s disease treatment-with-eating diet (CD-TREAT) is a prescriptive and personalised diet. It tries to recreate EEN as closely as possible using ordinary food by means of the exclusion of certain dietary components (e.g., gluten, lactose, and alcohol) and matching of others (macronutrients, vitamins, minerals, and fibre) [59]. The composition is based on Modulen IBD (Nestle, Vevey, Switzerland). Maltodextrin, an artificial glucose polymer and the commonest form of carbohydrate in EEN feeds, but not present in natural foods, is substituted by food high in starch and low in fibre. Because approximately 10% of food starch resists digestion and reaches the colon, it is hypothesized that this might influence the gut microbiome in a different way than EEN does. Therefore, the carbohydrate in CD-TREAT is decreased (particularly complex carbohydrates) in favour of protein. The micronutrients from EEN are accounted for by a multivitamin tablet. Next to providing the daily energy requirements, the CD-TREAT considers food preferences to increase palatability and adherence [59].

Svolos and colleagues assessed the efficacy of this dietary approach in a cross-over study including a metabolomic approach in 26 healthy adults and 5 children with active CD and compared CD-TREAT to EEN [59]. CD-TREAT was deemed easier to adhere to and more acceptable than EEN and induced similar effects on faecal microbiome composition, metabolome, and butyrate levels. In the children, after 4 weeks on CD-TREAT, 60% (3 of 5) clinically responded (weighted Paediatric Crohn’s disease activity index (wPCDAI) score change >17.5) and 40% (2 of 5) were in clinical remission (wPCDAI score <12.5). At 8 weeks, 80% (4 of 5) clinically responded and 60% (3 of 5) entered clinical remission. In the 4 children who completed 8 weeks of CD-TREAT, the wPCDAI score decreased significantly from baseline. One child dropped out due to symptom exacerbation [59]. These promising, translational results on clinical outcome and metabolomic endpoints warrant further confirmation in controlled and larger-scale trials.

### 3.6. IBD-AID (Inflammatory Bowel Disease-Anti-Inflammatory Diet)

The inflammatory bowel disease-anti-inflammatory diet (IBD-AID) diet consists of lean meats, poultry, fish, omega-3 fatty acids, eggs, particular sources of carbohydrate, select fruits and vegetables, nut and legume flours, limited aged cheeses (made with active cultures and enzymes), fresh cultured yogurt, kefir, miso and other cultured products (rich with certain probiotics), and honey [60]. Prebiotics, in the form of soluble fibre (containing beta-glucans and inulin, such as bananas, oats, blended chicory root, and flax meal) are suggested. In addition, trained dieticians support patients to begin at a texture phase of the diet matching with symptomology, starting with phase 1 (soft, well-cooked or pureed foods, no seeds) if in an active flare up to phase 4, when in remission and no structures are present. This is a complex diet with need of professional follow-up, but when implemented correctly, showed that in 11 IBD patients (8 CD) after 4 weeks all were able to downscale their medication regimen and all reported a reduction in IBD symptoms [60]. Thus, a large RCT seems warranted to show the true benefit.

### 3.7. FIT (Food Influence on the Intestinal Microbiota Diet)

The food influence on the intestinal microbiota diet (FIT) diet is a semi-vegetarian solid food diet characterized by the exclusion of added sugar and emulsifiers, increased consumption of fibre, and decreased consumption of meat and fish [61,62]. A pilot study including 28 healthy volunteers showed a manipulation of the microbiota and a significant decrease in faecal calprotectin [61,62]. Moreover, a significant decrease of serum IL-6 and Oncostatin M (OSM) in patients with ulcerative colitis after FIT diet was observed (unpublished), suggesting an anti-inflammatory mode of action. A larger RCT with the FIT diet in CD patients starting biological therapy is currently underway to evaluate its promising effect.

### 3.8. The Autoimmune Protocol Diet (AIP)/Paleolithic Diet

The autoimmune protocol (AIP) diet is an extension of the Paleolithic diet and consists of an initial elimination phase with avoidance of grains, legumes, nightshades, dairy, eggs, coffee, alcohol, nuts and seeds, refined/processed sugars, oils, and food additives, with the rationale that these foods and additives might trigger intestinal inflammation, dysbiosis, and/or symptomatic food intolerance [63]. The diet focuses on the elimination of gluten and refined sugar and emphasizes the consumption and preparation of fresh, nutrient dense foods, bone broth, and fermented foods. Special attention is given to life-style interventions such as sleep and sleep hygiene, stress management, forming a support system, and physical activity. After the elimination stage a reintroduction of food groups is initiated gradually, as patients identify unique foods or food groups that may contribute to symptoms while liberalizing their diet [63]. Konijeti and colleagues conducted a pilot study in 15 IBD patients (9 CD) with active disease and showed achievement of clinical remission as soon as 6 weeks after start of the study in 6 of the 9 CD patients which was maintained until the end of the study at 11 weeks (*p* < 0.01) [63]. There was a significant improvement in the Short Inflammatory Bowel Disease Questionnaire (SIBDQ) scores and general wellbeing in the 7 subjects that completed the study. The 2 remaining CD patients with ileal stricture dropped out because of worsening of symptoms (*n* = 1) and hospitalisation due to partial intestinal obstruction a few weeks after initiation of the elimination diet (*n* = 1). Overall, there was a trend for improvement of faecal calprotectin and mucosal healing, although only a subset of patients (of which only 2 with CD) received an endoscopic assessment. Therefore, although the AIP shows promise in improvement of symptoms and inflammation, the 2 adverse events mentioned, and the small sample size, preclude the use of this diet in a clinical setting. Large, randomized trials are needed to further investigate the value of this modified Paleolithic diet.

### 3.9. Mediterranean Diet

Besides multiple studies on cardiovascular disease, the Mediterranean diet has recently been studied for IBD patients as well. In the study by Chicco et al., the Mediterranean diet promoted the consumption of vegetables, fruits, breads and cereals, olive oil, legumes, fish/seafood, eggs, poultry, dairy foods, and low consumption of red meat and sweets. The study included 142 IBD patients (58 CD) and lasted for 6 months [64]. In the group of patients with CD, fewer patients on stable therapy had active disease. Furthermore, fewer patients had an elevated CRP, and faecal calprotectin and SIBDQ significantly decreased after the dietary intervention [64]. Further exploration of this possibly anti-inflammatory effect was recently provided by Lewis at all., comparing the Mediterranean to the SCD diet in CD patients. (DINE-CD, unpublished data) Although both diets were well tolerated and associated with clinical remission, this was not accompanied by normalization of CRP values.

### 3.10. (Semi-)Vegetarian Diet

The FACES trial randomized 214 CD patients in remission to low consumption of red or processed meat (defined as not more than 1 serving per month) or to a diet with a minimum of 2 servings/week of red or processed meat. No difference in relapse rates was observed between the diets [65]. However, compliance to the low consumption of red or processed meat was consistently lower during the whole trial, possibly explaining the negative results.

These results are contrasted by a prospective study by Chiba et al., where higher sustained remission rates were observed in patients on an semi-vegetarian compared to the control group [66]. However, the control group in this small non-randomized trial consisted of the patients refusing to follow the diet.

Further evidence supporting the lacto-ovo-vegetarian diet came from the same group showing high remission rates in adults with a new diagnosis (*n* = 26), children with a new diagnosis (*n* = 11), and relapsing adults (*n* = 9) with CD who were naïve to treatment with biologics when combining infliximab with the lacto-ovo-vegetarian diet [67]. After 6 weeks, 96% of patients following the diet achieved clinical remission on an intention-to-treat analysis and 100% per-protocol. A significant decrease in CDAI and CRP was observed after the dietary intervention. Moreover, mucosal healing was achieved in 46% (19/41) of cases [67]. These results suggest that exclusion of meat and/or fish to some extent might be a valuable component when designing a whole food diet for CD patients.

### 3.11. High-Fibre Diet

A comprehensive systematic review on the effect of fibre intake in IBD found no benefit on disease activity in any of the 12 studies in CD patients [25]. In contrast, although the primary endpoint was not met, several studies reported favourable intragroup effects on secondary outcomes including faecal butyrate, faecal calprotectin, inflammatory cytokines, microbiota, and gastrointestinal symptom indices [25], suggesting more research is needed to explain these contradictory findings.

In fact, Brotherton et al. found a significant improvement of health-related quality of life (*p* = 0.028) and gastrointestinal function (*p* = 0.008) in a small RCT when restricting refined carbohydrates and encouraging whole wheat bran through dietary advice. [68] Unfortunately, inflammatory markers did not differ between the two groups and the study population was extremely small (only 7 subjects) [68]. Lastly, the aforementioned trials studying the semi-vegetarian diet could be considered a high-fibre diet as well and has shown to be beneficial in CD patients in remaining remission and augmenting response to infliximab [66,67,69].

Importantly, despite earlier safety concerns in prescribing a high fibre diet in patients with CD, systematic reviews of RCT’s could not find evidence for restricting fibre intake besides in patients with overt obstructive symptoms [25].

### 3.12. Fat Content—Dairy Products

A large survey of 233 IBD patients reported that symptom onset and exacerbation were associated with dietary elements in 55% and 70% of patients, respectively [70]. Besides fruits and vegetables, frequently named ‘deleterious’ foods were dairy products, gluten-containing bread, and foods with a high fat content [70].

Interestingly, Nolan-Clark and colleagues reported in their survey of 156 CD patients that it was not the amount of lactose, but the fat content of dairy products that seemed to have a negative effect on symptoms [71].

A prospective study on the effect of dietary fat intake on relapse rates in 76 patients with inactive CD showed a worse prognosis when a decreased ratio of n-6 polyunsaturated fatty acid (PUFA) to n-3PUFA was consumed [72]. Conversely, moderate dietary temperance significantly decreased the risk of relapse [72].

Recent clinical trials assessing the effect of fat content on symptoms or inflammation in patients are scarce and the only data available assessed the effect of fat content or type of fat in different PEN and EEN formulas. In a systematic review of 29 clinical trials and 27 enteral feeds, there was only a significant positive correlation with response rate in feedings containing a high n-6 and n-3 fatty acids ratio (*p* = 0.018) [73]. A non- significant positive trend was found (*p* = 0.643) between medium chain triglycerides (MCT) delivery as a percentage of the total energy provision and remission rates. A non-significant negative trend was reported for the delivery of monounsaturated fatty acid (MUFA, *p* = 0.13) with a qualitative advantage to regimens based on safflower oil [73].

Taken together, the evidence concerning the type and quantity of fat intake on remission rates and symptoms in CD patients is low and conflicting, necessitating high quality, large clinical trials. The multicentre, randomized controlled EPIC-1 and -2 trials in 383 and 379 CD patients respectively, showed no effect of administration of omega-3 fatty acids in disease relapse prevention [74]. As stated by the authors, this type of evidence is important to guide patients into effective management trajectories, as the use of alternative medicine is widespread in this patient population [74,75].

### 3.13. Gluten-Free Diet

In a survey with 1647 IBD patients, 0.6% reported a concomitant diagnosis of celiac disease and 4.9% non-celiac gluten sensitivity. Nevertheless, as many as 19.1% had tried a gluten-free diet and 8.2% were currently consuming it. Improvement of gastrointestinal symptoms was reported in 65.6% of patients and fewer or less severe IBD flares in 38.3% [76].

Theoretically, gluten could create a pro-inflammatory environment in the intestine, leading to more frequent disease flares [77]. However, it cannot be excluded that a reduction or exclusion of gluten leads to a significant reduction of dietary fermentable oligo-, di-, monosaccharides and polyols (FODMAPs) and thereby reduces IBS-like symptoms in this IBD cohort [76,78]. This was illustrated in a double-blind crossover challenge in subjects with self-reported non-celiac gluten sensitivity, with induction of IBS-like symptoms after challenge with fructans, but not gluten [79]. More research is needed to elucidate this important difference and assess if reduction or exclusion of gluten indeed results in a reduced inflammatory status.

### 3.14. Low-FODMAP (Fermentable Oligo-, Di-, Monosaccharides and Polyols) Diet

The low-FODMAP diet specifically limits fructose, lactose, fructans, galactans and polyols and has shown effectiveness in improving symptoms of irritable bowel syndrome (IBS) [15,80]. Importantly, the low-FODMAP diet has not been developed to reduce intestinal inflammation. The theoretical base is partially similar to that of the SCD in that it tries to exclude poorly absorbed short-chain carbohydrates, that can be fermented by intestinal bacteria resulting in gas and bloating, abdominal pain and change in bowel habits [15]. Additionally, FODMAPs are osmotically active and can lead to more fluid delivery to the colon.

It is estimated that 35% of patients with IBD experience gut symptoms despite having quiescent disease [81,82]. The aetiology of these gut symptoms is hypothesized to relate to visceral hypersensitivity due to previous gastrointestinal inflammation, persistent unidentified low-grade inflammation or the psychological impact of IBD [81,83]. Of course, since IBS is highly prevalent in the general population, concomitant IBS in IBD patients is to be expected in 5–10% of patients [84].

Because symptomatic response to therapy might depend on treating the IBS-component in IBD and not on any anti-inflammatory effect, research has aimed to explore the efficacy of a low-FODMAP diet in an IBD-population. An Australian pilot study assessed the low-FODMAP diet in CD and UC patients using a retrospective telephone questionnaire for IBD patients with coexisting functional gut symptoms. This study showed an improvement in symptoms of those who responded, particularly in abdominal pain, bloating, flatulence, and diarrhoea. Importantly, inflammatory markers were not assessed [85,86].

Cox et al. performed a cross-over and rechallenge RCT in 28 patients with inactive IBD and functional gastrointestinal symptoms challenged 3 days with galacto-oligosaccharides, fructan, sorbitol and glucose (placebo) with a 4-day washout period after each intervention, and reported a significant worsening of gastrointestinal symptoms only after (again) fructan ingestion. A worrisome finding was that although no significant differences in CRP were measured, the faecal calprotectin levels in CD patients were significantly elevated at the end of the trial [87]. A larger RCT from the same group 3 years later implemented a 4-week low-FODMAP diet vs. sham diet in 52 patients and showed a significantly better symptom relief and health-related quality of life scores [81]. Given the concern about safety, a 6-week randomized trial in 55 IBD patients (of whom 38 suffered CD) comparing a low-FODMAP to a standard diet assessing safety and efficacy, showed a significant reduction in HBI and faecal calprotectin in CD patients and a slight and barely significant improvement of quality of life scores [88]. There was no significant change in CRP levels. Although this study was designed to assess safety, the short-term nature and relatively small numbers of the study precludes definite conclusions on long-term use of a low-FODMAP diet.

However, the question remains if amelioration in gastrointestinal symptoms translates into IBD-specific therapeutic targets such as clinical and endoscopic remission. To our knowledge, there is no evidence to date that a low-FODMAP diet reduces the inflammatory burden in IBD-patients. Notably, the low-FODMAP diet excludes inulin, fructo-oligosaccharides and fructose, which are known prebiotics. This could potentially translate in an exacerbation of the dysbiosis in CD [15]. Recent evidence to support this hypothesis is emerging with RCT’s demonstrating a reduction in concentration and proportion of luminal Bifidobacteriae in a UK cohort [89] and a reduction of total bacterial abundance [90] in an Australian RCT using the low-FODMAP diet [89,90]. The aforementioned RCT from Cox et al. reported a significantly lower abundance of *Bifidobacterium adolescentis, Bifidobacterium longum,* and *Faecalibacterium prausnitzii* than in patients on control diet [81]. Furthermore, the Australian study showed a lower relative abundance of *Akkermansia muciniphila* and a higher relative abundance in *Ruminococcus torques*, a pattern that was similar to the difference seen in IBD patients vs. controls [90,91,92]. Although the functional significance and health implications of such changes are unknown, reducing FODMAP intake in the longer term should be implemented with caution, certainly in an IBD population with known dysbiosis and alterations of the mucosal barrier [90,93].

A low-FODMAP diet might be helpful in controlling IBS symptoms, but should only be considered when implemented with caution and dietary advice, due to the restrictive nature of the diet. Furthermore, dosing the amount of FODMAPs to the individual to limit the restriction as much as possible should be pursued. This limitative restriction is paramount due to the lack of inflammatory benefit and potential detrimental effects on microbial diversity, mucus layer and overall nutritional status.

### 3.15. Other Diets

After a pilot study with 5 patients, Bartel et al. conducted an RCT with 18 mild-to-moderate CD patients comparing a very restrictive exclusion diet vs. a low-fat, high-carbohydrate diet, excluding fibre-rich fruits and vegetables and red meat [94]. The former group was allowed to eat red meat, certain sourdough bread, rapeseed oil, and fresh butter (as spread for bread), all of which came from intensively monitored organic farming. Only plain tap water and organic tea were allowed for drinking, and rock salt (halite) was allowed for spicing. No fruits or vegetables were permitted. Baking soda toothpaste was provided, and the subjects must limit dish washing (only water). 4 patients dropped out of the study (3 in the intervention group). At 6 weeks magnetic resonance imaging (MRI) and endoscopy showed improvement of intestinal lesions in 3 of 4 assessable patients of the active group and 1 of 9 patients of the control group (*p* = 0.027). In contrast, CDAI and IBDQ improved in both groups to a similar extent [94]. Although a positive result, the diet seems extremely restrictive and hard to implement in real life, limiting its acceptability.

A 4-arm, single blinded RCT by Lomer et al. involving 82 patients with active CD on a diet with either a low or high calcium content and low or high microparticle content after induction with prednisolone could not show a significant difference in remission rates or clinical response [95].

Komperod et al. found in a small non-controlled prospective cohort of 12 patients with CD in remission that a personalised exclusion diet of self-reported dietary triggers of 2 weeks after a habitual diet resulted in significant improvement of gastrointestinal symptoms [96]. Important to note is that inflammatory markers were not assessed, limiting its therapeutic potential.

## 4. Conclusions

The beneficial effects of EEN and PEN have long been established in both paediatric and adult Crohn’s disease, but the difficulty in long-term adherence due to taste fatigue and social incompatibility has made it a largely unacceptable tool in long-term management. In contrast, an ideal dietary intervention should be palatable, have a high chance of long-term adherence, is acceptable and compatible with a social/professional life and has a biologically plausible mode of action, backed up by a strong evidence base.

Unfortunately, many difficulties arise in understanding how EEN achieves its results: is it the exclusion of harmful elements in our habitual diet or is it the addition of beneficial substances that explains its success? Additionally, known problems in dietary research with difficulties in assessing compliance to the tested dietary strategy and cumbersome implementation of a true control group persist until today. This results in a very heterogeneous study landscape, complicating comparing results as illustrated by this narrative review. Despite these hurdles, major progress has been made in the development of several whole food diets with variable scientific back-up showing its efficacy when used as a monotherapy or in combination in both induction and maintenance of remission in CD. Today however, large RCTs assessing the efficacy of the aforementioned diets on mucosal healing with the scientific rigor that applies to pharmacological substances are lacking and desperately needed to guide future clinical practice.

## Figures and Tables

**Table 1:** Short description of dietary interventions.

Diet	Intervention	Personalized
EEN	Liquid formula-based diet delivered to the gastro-intestinal tract orally or through nasogastric feeding.	No
PEN	Whole food diet supplemented with liquid formula-based diet for a prespecified percentage of calories.	No
SCD	Restriction of complex carbohydrates and elimination of refined sugar.	No
IgG-guided exclusion	Exclusion of foods with high serum anti-IgG titers.	Yes
CDED	Exclusion of gluten and gluten-free baked goods, dairy products, animal fat, processed meats, emulsifiers, canned goods, and all packaged products with a due date. Restrictions can be loosened after 6 weeks.	No
CD-TREAT	Exclusion of gluten, lactose, and alcohol and matching of macronutrients, vitamins, minerals, and fibre with ordinary foods and multivitamin tablets, guided by personal preference.	Yes
IBD-AID	Whole foods diet consisting of lean meats, poultry, fish, omega-3 fatty acids, eggs, particular sources of carbohydrate, select fruits and vegetables, nut and legume flours, limited aged cheeses (made with active cultures and enzymes), fresh cultured yogurt, kefir, miso and other cultured products, and honey. Probiotics are suggested and dietetic advice is necessary for the symptomatology-based texture adaptations through the course of the diet.	Yes
FIT	Semi-vegetarian diet characterized by the exclusion of added sugars, processed foods, and emulsifiers, increased consumption of fibre, and decreased consumption of meat and fish.	No
AIP	Avoidance of grains, legumes, nightshades, dairy, eggs, coffee, alcohol, nuts and seeds, refined/processed sugars, oils, and food additives with personalized reintroduction combined with life-style advice.	Yes
Mediterranean	Diet promoting consumption of vegetables, fruits, breads and cereals, olive oil, legumes, fish/seafood, eggs, poultry, dairy foods, and low consumption of red meat and sweets.	No
Semi-Vegetarian	Low consumption of red or processed meat (not more than 1 serving per month).	No
Lacto-Ovo-(Semi)vegetarian	Consumption of eggs, milk, vegetables, legumes, fruits, rice, soup, potatoes and plain yoghurt. (Only one serving of meat per 2 weeks, 1 serving of fish per week.)	No
Gluten-free	Strict exclusion of gluten in the diet.	No
Low-FODMAP	Diet limiting fructose, lactose, fructans, galactans and polyols, thereby excluding poorly absorbed short-chain carbohydrates.	No
Low-Microparticle	Limiting of foods with microparticles TiO_2_ and/or Psil.	No

EEN: exclusive enteral nutrition, PEN: partial enteral nutrition, SCD: specific carbohydrate diet, IgG: Immunoglobulin G, CDED: Crohn’s disease exclusion diet, CD TREAT: Crohn’s disease Treatment-with-Eating diet, IBD-AID: Inflammatory Bowel Disease Anti-Inflammatory Diet, FIT: Food Influence on the Intestinal Microbiota diet, AIP: Auto-Immune Protocol diet, FODMAP: fermentable oligo-, di-, monosaccharides and polyols.
